# Nomogram for the prediction of triple-negative breast cancer histological heterogeneity based on multiparameter MRI features: A preliminary study including metaplastic carcinoma and non- metaplastic carcinoma

**DOI:** 10.3389/fonc.2022.916988

**Published:** 2022-09-20

**Authors:** Qing-cong Kong, Wen-jie Tang, Si-yi Chen, Wen-ke Hu, Yue Hu, Yun-shi Liang, Qiong-qiong Zhang, Zi-xuan Cheng, Di Huang, Jing Yang, Yuan Guo

**Affiliations:** ^1^ Department of Radiology, The Third Affiliated Hospital, Sun Yat-Sen University, Guangzhou, China; ^2^ Department of Radiology, Guangzhou First People’s Hospital, School of Medicine, South China University of Technology, Guangzhou, China; ^3^ Breast Tumor Center, Sun Yat-sen Memorial Hospital, Sun Yat-sen University, Guangzhou, China; ^4^ Department of Pathology, Guangzhou First People’s Hospital, School of Medicine, South China University of Technology, Guangzhou, China; ^5^ Department of Breast Surgery, Guangzhou First People’s Hospital, School of Medicine, South China University of Technology, Guangzhou, China

**Keywords:** nomograms, triple negative breast cancer, magnetic resonance imaging, metaplastic breast carcinoma, histological subtypes

## Abstract

**Objectives:**

Triple-negative breast cancer (TNBC) is a heterogeneous disease, and different histological subtypes of TNBC have different clinicopathological features and prognoses. Therefore, this study aimed to establish a nomogram model to predict the histological heterogeneity of TNBC: including Metaplastic Carcinoma (MC) and Non-Metaplastic Carcinoma (NMC).

**Methods:**

We evaluated 117 patients who had pathologically confirmed TNBC between November 2016 and December 2020 and collected preoperative multiparameter MRI and clinicopathological data. The patients were randomly assigned to a training set and a validation set at a ratio of 3:1. Based on logistic regression analysis, we established a nomogram model to predict the histopathological subtype of TNBC. Nomogram performance was assessed with the area under the receiver operating characteristic curve (AUC), calibration curve and decision curve. According to the follow-up information, disease-free survival (DFS) survival curve was estimated using the Kaplan-Meier product-limit method.

**Results:**

Of the 117 TNBC patients, 29 patients had TNBC-MC (age range, 29–65 years; median age, 48.0 years), and 88 had TNBC-NMC (age range, 28–88 years; median age, 44.5 years). Multivariate logistic regression analysis demonstrated that lesion type (p = 0.001) and internal enhancement pattern (p = 0.001) were significantly predictive of TNBC subtypes in the training set. The nomogram incorporating these variables showed excellent discrimination power with an AUC of 0.849 (95% CI: 0.750−0.949) in the training set and 0.819 (95% CI: 0.693−0.946) in the validation set. Up to the cutoff date for this analysis, a total of 66 patients were enrolled in the prognostic analysis. Six of 14 TNBC-MC patients experienced recurrence, while 7 of 52 TNBC-NMC patients experienced recurrence. The DFS of the two subtypes was significantly different (p=0.035).

**Conclusions:**

In conclusion, we developed a nomogram consisting of lesion type and internal enhancement pattern, which showed good discrimination ability in predicting TNBC-MC and TNBC-NMC.

## Introduction

Breast cancer is a heterogeneous disease. Over the past 40 years, the American Joint Commission on Cancer (AJCC) has refined and improved the anatomical classification of breast cancer, gradually adding pathological and molecular biological factors to reflect the complex biological characteristics of breast cancer. The heterogeneity of breast cancer is related to molecular subtypes and histological features. Among the molecular subtypes of breast cancer, triple negative breast cancer (TNBC) has different histological characteristics and is associated with high invasive proliferation and low differentiation (1).

The histologic heterogeneity in TNBC significantly informs patient outcomes (2), among which, metaplastic carcinoma (MC) has been a focus because of its specific clinical and pathological features compared with non-metaplastic carcinoma (NMC) (3–5). Previous studies have shown that TNBC-MC is less sensitive to adjuvant therapy and has a poorer prognosis than TNBC-NMC in the same clinical stage, indicating that it is necessary to develop optimum personalized treatments for these two different histological characteristics (6–8).

The accurate diagnosis of patients with TNBC-MC or TNBC-NMC remains a challenge due to less experience (8). However, early diagnosis followed by personalized treatment is critical to improving the poor survival rates. Therefore, it is particularly important to improve the accurate assessment for TNBC-MC and TNBC-NMC. Among mammography, ultrasound and magnetic resonance imaging (MRI), MRI is widely applied to obtain an accurate preoperative diagnosis of breast tumors because of its high resolution and multiple parameters (9). MRI can present the lesion and boundaries of the tumor (10). A few reports have discussed the morphological characteristics of MC based on MRI (10–13); however, the number of patients was relatively small (14), previous studies explored single imaging characteristics, and few have developed predictive models using combined imaging characteristics. Hence, it is necessary to build an effective preoperative model for predicting the TNBC histological heterogeneity.

Nomograms, which are easy-to-use graphical predictive tools, have been widely applied to predict numerous binary and prognostic outcomes (15). In previous studies, nomograms have provided detailed probabilities of different clinical events and helped clinicians make decisions in the management of breast cancer (16, 17). However, there seems to be no nomogram based on MRI features for the prediction of TNBC-MC and TNBC-NMC in the literature. Therefore, the purpose of this study was to develop and validate a nomogram for the preoperative prediction of TNBC histological heterogeneity based on multiparameter MRI features.

## Materials and methods

### Patients

**Figure 1:**
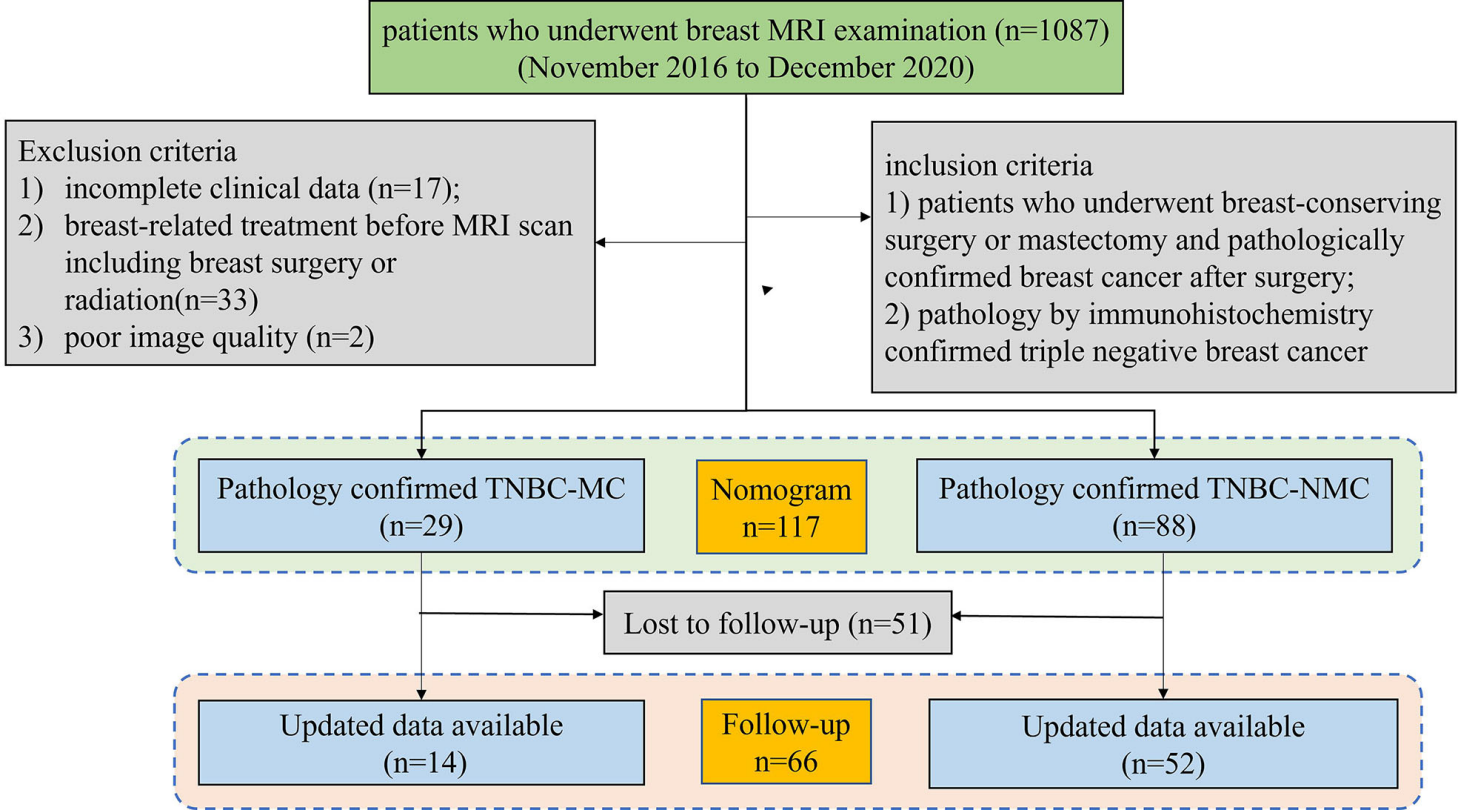
Flowchart of the patient selection and exclusion criteria. MRI, magnetic resonance imaging. TNBC;MC; triple negative breast cancer-metaplastic carcinoma; TNBC-NMC, triple negative breast cancer- non metaplastic carcinoma.

This retrospective study was approved by the Ethics Committee of the Second Affiliated Hospital of South China University of Technology (K-2019-023-01). The patients provided written informed consent to participate in this study. A total of 1087 patients between November 2016 and December 2020 underwent multiparameter MRI for preoperative assessment, initial staging, neoadjuvant chemotherapy (NAC) response evaluation, follow-up, and screening of a high-risk population. Finally, 117 patients (including 29 MC patients and 88 NMC patients) were confirmed to have TNBC by immunohistochemistry pathology. The inclusion criteria were as follows: (1) patients who underwent breast-conserving surgery or mastectomy and had pathologically confirmed breast cancer after surgery; and (2) patients confirmed with TNBC by immunohistochemistry. The exclusion criteria included (1) incomplete clinical data (n = 17); and (2) breast-related treatment before MRI scans, including breast surgery or radiation (n = 33); (3) poor image quality (n=2). Then, the patients were randomly split into a training set and a validation set for model validation in a 3:1 ratio. Among them, 66 patients had follow-up information. **Figure 1** shows the patient inclusion process.

### MRI examination

The MRI scans were performed from three hospitals on a 1.5 T MRI system (Philips, Achieva Systems, Netherlands; Siemens, Magnetom Avanto, Germany; United Imaging, uMR 560, China) or a 3.0 T MRI system (Siemens, Magnetom Skyra, Germany) with a breast coil. The sequence scanning parameters were as follows: axial T1-weighted imaging (T1WI) and fat-suppressed T2-weighted imaging (T2WI) images; gadolinium (Gd-DTPA, Magnevist; Bayer Healthcare, Berlin, Germany) was used as the contrast enhancement agent at a dose of 0.2 ml/kg body weight and a rate of 1.5 ml/s; and axial 3D fat-saturated T1WI was performed after injection. The detailed acquisition parameters are shown in **Table 1**.

**Table 1:** Breast MRI sequences and acquisition parameters in three centers.

Hospital	Scanner	Sequence	TR(ms)	TE(ms)	FOV(mm^2^)	Slice thickness (mm)	Interslice gap (mm)	Acquisition time (sec)
Center 1	1.5-T MRI system (uMR 560, United Imaging)	T1WI+C	5.1	2.1	320×320	2.4	0.48	394
		T1WI	4.8	2.1	320×320	3	0.3	78
		T2WI	3800	42.7	328×350	4	0.8	126
	3.0-T MRI system (Siemens, Skyra)	T1WI+C	4.67	1.66	360×360	1.5	0	333
		T1WI	6.05	2.46	340×340	1.5	0	45
		T2WI	3500	79	340×340	4	0.4	219
Center 2	1.5-T MRI system(Siemens, Germany)	T1WI+C	4.87	2.4	380×380	3	1.5	322
		T1WI	6.86	2.39	360×360	2	0.4	139
		T2WI	2550	107	350×350	5	1	153
Center 3	1.5-TMRI System (Philips, Netherlands)	T1WI+C	7.0	3.4	340×340	3	0	568
		T1WI	535	10	340×340	3	0	169
		T2WI	4121	120	340×340	3	0	62

### MR image analysis

For evaluation of tumor morphology, all MR images were independently reviewed by two breast radiologists (14 years and 8 years of experience), according to the fifth edition of the Breast Imaging Reporting and Data System atlas (18). When the analysis results were inconsistent, the case was discussed with each other for determination by consensus. Both were aware that study participants had breast cancer, but they were blinded to the rest of the histopathologic results.

The size of tumors, T1WI signal (hyperintense or hypo&isointense), T2WI signal (iso&hypo intense or hyperintense), lesion type (mass or nonmass&both), shape (round&oval or irregular), margin (circumscribed or not) and internal enhancement pattern (heterogeneous or rim enhancement) were assessed. According to the Breast Imaging Reporting and Data System Lexicon (BI-RADS), nonmass enhancement(NME) represents an area of contrast enhancement without a space-occupying effect and mass enhancement in three dimensional space has fat pushing effect (19). As for internal enhancement pattern, we mainly refer to the our previous study which defined rim enhancement: more pronounced contrast enhancement at the periphery of a tumor compared to that at the center and heterogeneous enhancement: with heterogeneous enhancement in the tumor (20).

### Pathology

Histological features were determined from surgical resection specimens, including expression of ER, PR, HER-2 and Ki-67 and grade of differentiation. 29 tumors showed TNBC-MC (including 17 squamous carcinomas, 7 spindle cell, 5 with mesenchymal differentiation matrix producing), 88 tumors showed TNBC-NMC (including 61 invasive breast carcinomas of no special type (IBC-NST), 22 IBC-NST+ ductal carcinoma *in situ* (DCIS), 3 adenoid cystic carcinoma and 2 medullary carcinoma).

### Patient follow-up

Follow-up of 66 patients (before 30 Sep 2018) in the whole cohort was completed until 30 Sep 2021. Follow-up information was acquired through a review of the electronic medical records, and the duration of follow-up was calculated as the elapsed period between the date of surgery and the last date of follow-up, occurrence of any event, or death. Disease-free survival (DFS) events were defined as follows: the first recurrence of invasive breast cancer at a local, regional, or distant site; the incidence of contralateral breast cancer; and death from any cause. Patients without DFS events were censored at the last follow-up.

### Statistical analysis

Statistical analysis was performed using SPSS 23.0 (Chicago, USA). Interobserver reliability between two radiologists was assessed using interclass correlation coefficient. An ICC value of 1.0 was deemed to indicate perfect agreement; 0.81–0.99, almost perfect agreement; 0.61–0.80, substantial agreement; 0.41–0.60, moderate agreement; 0.21–0.40, fair agreement; and ≤ 0.20, slight agreement (21). The training set and validation set results were compared using the χ2 test or Fisher’s exact test. Risk factors were identified by univariate analysis based on the χ2 test or Fisher’s exact test, and the statistically significant variables (p<0.05) were included in the final models by applying multivariate logistic regression to the training set. Nomogram construction and cumulative survival analysis were performed using R version 3.6.3 and Python version 3.7. A ROC curve was used to evaluate the sensitivity and specificity of the nomogram prediction model. Calibration curves were plotted to assess the calibration of the nomogram. P values ≤0.05 were considered statistically significant. The statistical analyses were similar to those performed in our previously published study (22).

## Results

### Patient characteristics

A total of 29 patients with TNBC-MC (age range, 29–65 years; median age, 48.0 years) and 88 with TNBC-NMC (age range, 28–88 years; median age, 44.5 years) were included in this study. A total of 117 patients were divided into the training cohort and the validation cohort at an approximate ratio of 3 to 1. There were no significant differences in type, age, location, symptom, menopausal status, pathological grade, axillary lymph node (ALN) status, ductal carcinoma *in situ* (DCIS) status or Ki-67 status between the training cohort and the validation cohort (all p > 0.05). The clinical and pathological characteristics of the 117 patients are listed in **Table 2**.

**Table 2:** Patient clinical and pathological information of MC and NMC in training set and validation set.

	Training set (n=88)	Validation set (n=29)	P value
Type			
TNBC-NMC	68 (77.3%)	20(69.0%)	0.515
TNBC-MC	20(22.7%)	9 (31.0%)	
Age	48.41±13.54	46.34±10.76	0.698
Menopausal status			0.948
Premenopausal	52 (59.1%)	18 (62.1%)	
Postmenopausal	36 (40.9%)	11 (37.9%)	
Symptoms			0.354
Breast lumps	70 (79.5%)	20 (69.0%)	
Pain	16 (18.2%)	8 (27.6%)	
Other	2 (2.3%)	1 (3.4%)	
Location			0.558
L	50 (56.8%)	14 (48.3%)	
R	38 (43.2%)	15 (51.7%)	
Pathological grade			0.557
2	53 (60.2%)	15 (69.0%)	
3	35 (39.8%)	14 (31.0%)	
Axillary lymph node			1.000
negative	55 (62.5%)	18 (62.1%)	
positive	33 (37.5%)	11 (37.9%)	
DCIS present			0.542
No	74 (84.1%)	26 (89.7%)	
yes	14 (15.9%)	3 (10.3%)	
Ki-67			0.103
<20	4 (4.5%)	4 (13.8%)	
≥20	84 (95.5%)	25 (86.2%)	

DCIS, ductal carcinoma *in situ;* TNBC,triple-negative breast cancer; MC, metaplastic carcinoma; NMC, non-metaplastic carcinoma.

### MRI features of TNBC-MC and TNBC-NMC in the training and validation sets

For the MRI features, the ICC analysis showed a good agreement among the two readers with ICC values ranging from 0.813–0.872. Patients in the TNBC-MC group had more rim enhancement and less nonmass enhancement & both than those in the TNBC-NMC group, and the differences were statistically significant in the training set (p= 0.029 and p=0.001, respectively). The MRI features of TNBC-MC and TNBC-NMC patients in the training and validation sets are detailed in **Table 3**.

**Table 3:** MRI features of TNBC-MC and TNBC-NMC in the training and validation set.

	Training set (n=88)			Validation set (n=29)		
	NMC (n=68)	MC (n=20)	P value	NMC (n=20)	MC (n=9)	P value
Size (mm)			0.269			0.143
≤20	18 (26.5%)	2 (10.0%)		5 (25.0%)	1(11.2%)	
>20and≤50	36 (52.9%)	14 (70.0%)		13(65.0%)	4 (44.4%)	
>50	14 (20.6%)	4 (20.0%)		2 (10.0%)	4 (44.4%)	
T1WI			0.075			0.287
Hyperintense	4 (5.9%)	4 (20%)		2 (10%)	3 (33.3%)	
Hypo or isointense	64 (94.1%)	16 (80%)		18 (90%)	6 (66.7%)	
T2WI			0.318			1.000
Iso or hyper intense	65 (95.6%)	18 (80.0%)		18 (90.0%)	9(100%)	
Hypointense	3 (4.4%)	2 (20.0%)		2 (10.0%)	0	
Lesion type			**0.001**			0.066
Mass	36 (52.9%)	19 (95.0%)		13 (65.0%)	9 (100%)	
Nonmass & both	32 (47.1%)	1 (5.0%)		7 (35.0%)	0 (0.0%)	
Shape			0.543			1.000
Round/oval	19 (27.9%)	7 (35.0%)		8 (40.0%)	4 (44.4%)	
Irregular	49 (72.1%)	13 (65.0%)		12 (60.0%)	5 (55.6%)	
Margin			0.481			0.287
Circumscribed	9 (13.2%)	4 (20.0%)		2 (10.0%)	6 (66.7%)	
Not clear	59 (86.8%)	16 (80.0%)		18 (90.0%)	3 (33.3%)	
Internal enhancement pattern			**0.029**			0.209
Heterogeneous	50 (73.5%)	9 (45.0%)		16 (80.0%)	5 (55.6%)	
Rim enhancement	18 (26.5%)	11 (55.0%)		4 (20.0%)	4 (44.4%)	

TNBC,triple-negative breast cancer; MC, metaplastic carcinoma; NMC, non-metaplastic carcinoma. Bold means P values ≤0.05 were considered statistically significant.

### Univariable and multivariable logistic regression analyses for predicting TNBC-MC and TNBC-NMC

For the clinicopathological factors, there was no significant difference in age, location, menopausal status, ALN status and DCIS between the TNBC-MC and TNBC-NMC groups; in addition, the proportion of high pathological grade (pathological grade 3) for TNBC-MC and TNBC-NMC was 65% and 32.4%, respectively, with a significant difference between two groups (p = 0.019). For the MR factors, there were significant differences in lesion type (p=0.007) and internal enhancement pattern (p = 0.020) between the TNBC-MC and TNBC-NMC groups. Then the significant variables of univariate logistic regression were included in multivariate logistic regression. Multivariate logistic regression analysis demonstrated that lesion type (p = 0.001) and internal enhancement pattern (p = 0.001) were significantly predictive of TNBC subtypes (**Table 4**).

**Table 4:** Univariate and multivariable logistic analysis of clinical, MR and pathological features for predicting TNBC subtypes in the training set.

Variable	No.		Univariate analysis		Multivariate analysis	
			OR (95%CI)	P value	OR (95%CI)	P value
Clinicopathologic factors						
Age (years)			1.003 (0.967,1.041)	0.853		
Axillary lymph node			0.336 (0.102,1.112)	0.074		
Negative	55	Ref				
Positive	33					
Menopausal status			1.242 (0.454,3.398)	0.672		
Premenopausal	52	Ref				
Postmenopausal	36					
Location			1.429 (0.525,3.886)	0.485		
L	50	Ref				
R	38					
Pathological grade			3.883 (1.359,11.096)	**0.019**	1.776 (0.444, 7.098)	0.417
2	53	Ref				
3	35					
DCIS present			0.223 (0.027,1.818)	0.161		
No	74	Ref				
yes	14					
MR factors						
Size (mm)						
≤20	20	Ref				
>20and≤50	50		0.389 (0.062,2.438)	0.313		
>50	18		1.361 (0.382,4.852)	0.635		
T2WI			0.415 (0.064,2.678)	0.356		
Hypo or isointense	83	Ref				
Hyperintense	5					
T1WI			4.000 (0.901,17.753)	0.068		
Hypo or isointense	80	Ref				
Hyperintense	8					
Shape			0.720 (0.249,2.080)	0.544		
Round/oval	26	Ref				
Irregular	62					
Lesion type			0.059 (0.007,0.468)	**0.007**	0.009 (0,0.147)	**0.001**
Mass	55	Ref				
Nonmass & both	33					
Internal enhancement pattern			3.395 (1.209,9.535)	**0.020**	37.197 (4.210,328.681)	**0.001**
Heterogeneous	59	Ref				
Rim enhancement	29					

DCIS, ductal carcinoma *in situ;* TNBC, triple-negative breast cancer. P values ≤ 0.05 were considered statistically significant. Bold means P values ≤0.05 were considered statistically significant.

### Development and performance of the nomogram

**Figure 2:**
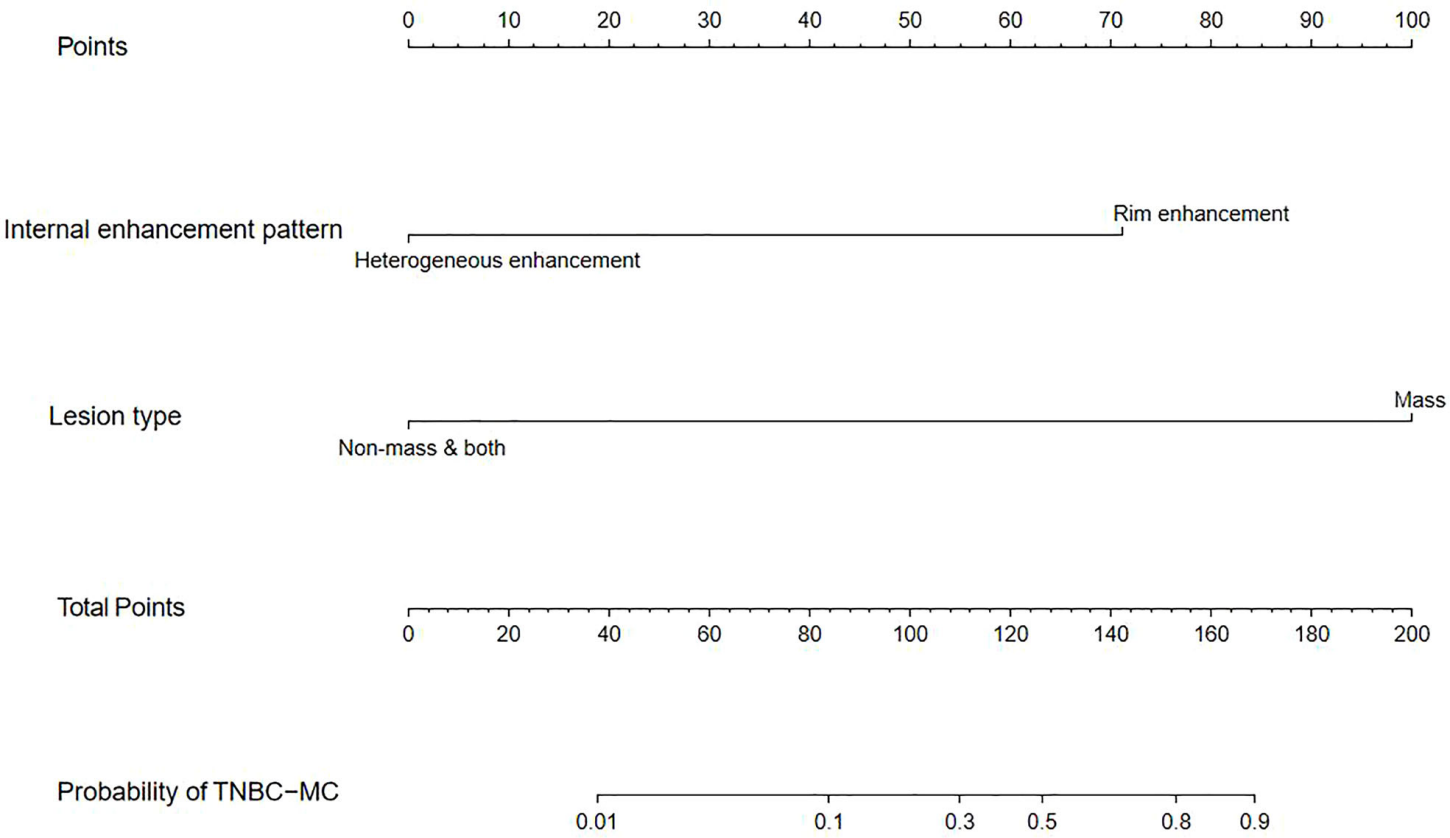
Nomogram constructed based on the combined model.

We constructed a nomogram based on the statistically significant variables identified in the multivariate logistic regression analysis, including lesion type and internal enhancement pattern (**Figure 2**). Each variable is assigned a point value by drawing a vertical line between the appropriate variable value and the scale in the first row. The total nomogram score is calculated by summing the scores for each of the variables. Then, the probability of TNBC subtypes can be determined by drawing a vertical line between the total score and the scale in the last row.

**Figure 3:**
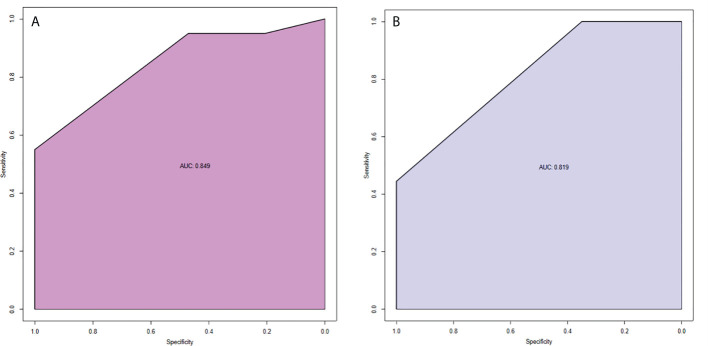
ROC curves of nomogram in the training set **(A)** and validation set **(B)**. AUC of the predictive model was 0.849 (95% CI = 0.750−0.949). AUC of the model of the validation set was 0.819 (95% CI = 0.693−0.946).

**Figure 4:**
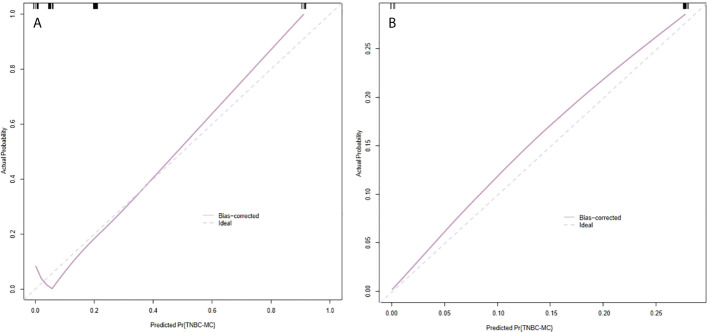
Calibration slope of nomogram in the training set **(A)** and validation set **(B)**. The calibration curve was used to represent the relationship between the predicted value and the true value. The x-axis represented the probability of TNBC-MC predicted by the nomogram, and the y-axis represented the actual TNBC-MC rate. The blue line in the middle represented the accurate prediction. And the purple line represented the predictive power of the nomogram. The closer the purple line and the blue line were, the better the prediction effect of the nomogram. Both p value >0.05 in the training and validation sets. TNBC-MC, triple negative breast cancer-metaplastic carcinoma.

**Figure 5:**
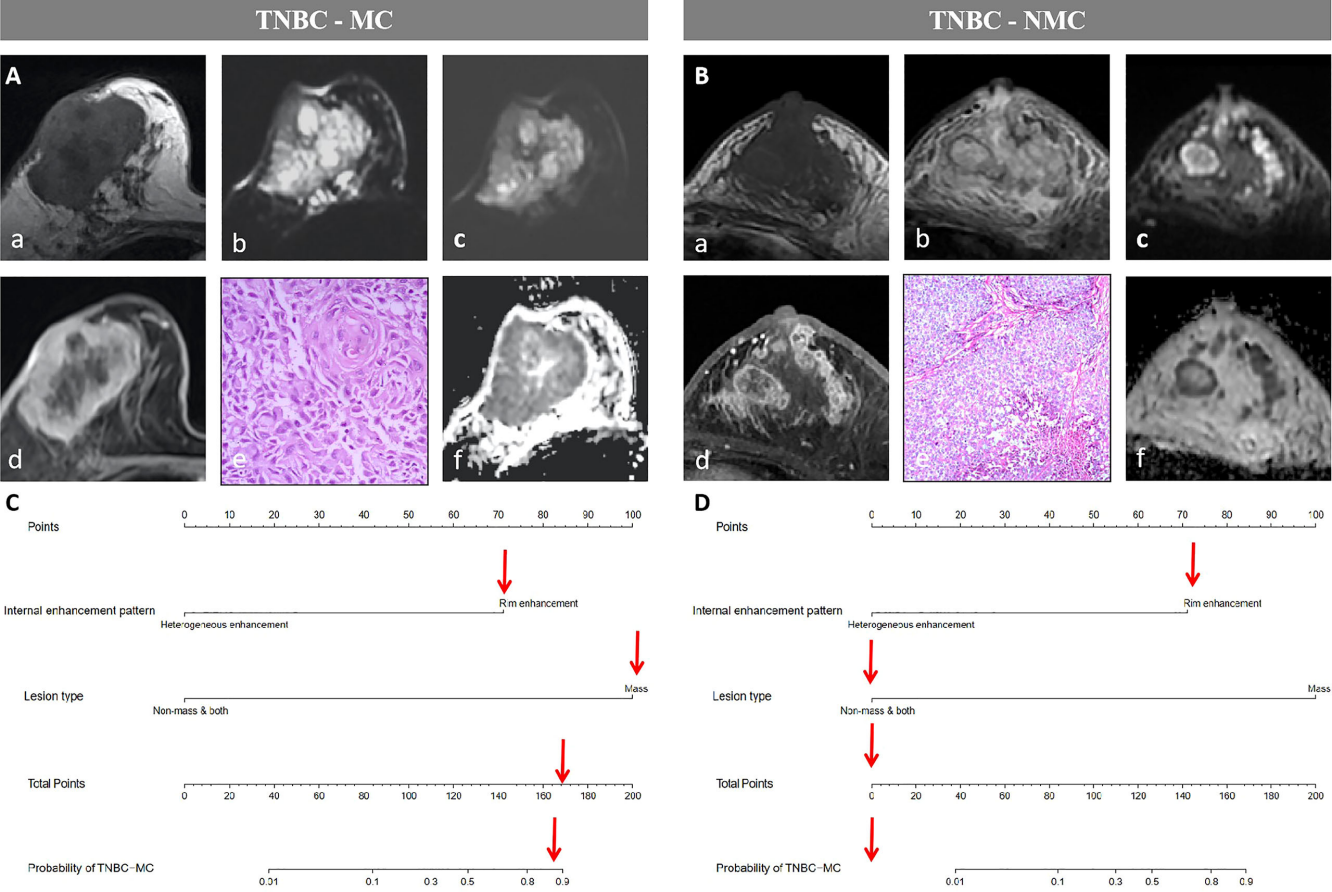
Examples of the nomogram in use. **(A)** Breast MRI examination of a 59-year-old TNBC-MC patient. A(a) The lesion showed iso- or hypo- intense signal on T1WI. (b)T2WI showed the lesion with hyperintense signal on T2WI. (c) Diffusion-weighted imaging (DWI) showed inhomogeneous iso- or hyper- intense signal. (d) The lesion showed mass type with circumscribed margin, and rim enhancement. (e) HE staining (×40), squamous cell carcinoma be observed under microscope. (f) Apparent diffusion coefficient (ADC) showed hypointense. **(B)** Breast MRI examination of a 61-year-old TNBC-NMC patient. B(a) The lesion showed hypo- intense signal on T1WI. (b)T2WI showed the lesion with iso- or hyper- intense signal on T2WI. (c) DWI showed significantly inhomogeneous iso- or hyper- intense signal. (d) The lesion showed both type (mixed type with mass and nonmass) with not clear margin, and heterogeneous enhancement. (e) HE staining (×10), non-invasive carcinoma be observed under microscope. (f) ADC showed significantly inhomogeneous hypointense. **(C)** The possibility of MC assessed by nomogram could be more than 80%. **(D)** The possibility of MC assessed by nomogram could be less than 10%.

**Figure 6:**
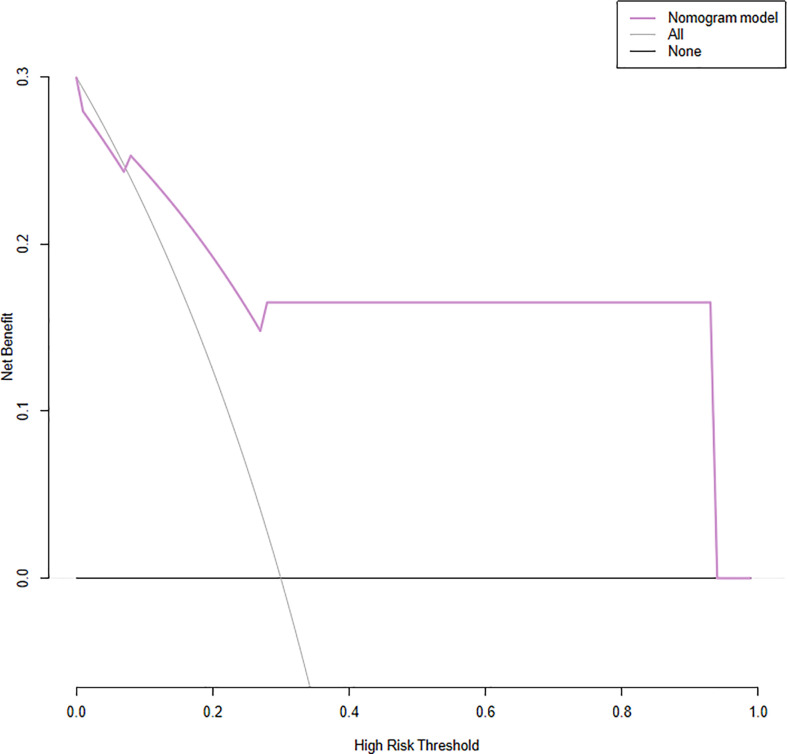
Decision curve analysis (DCA) for the nomogram. The y-axis represents the net benefit. The x-axis represents the threshold probability, which means that the expected benefit of treatment is equivalent to the expected benefit of non-treatment, the decision curve shows that the nomogram model (purple line) adds more benefit than either the treat-all scheme (grey line) or treat-none scheme (black line) in predicting sentinel lymph node burden when the threshold probability ranges from 16 to 96%.

The model had good sensitivity and specificity, with an AUC of 0.849 in the training set (**Figure 3A**). A calibration graph was drawn with the predicted probability of TNBC-MC and TNBC-NMC (**Figure 4A**). To validate the nomogram’s stability, we also conducted a validation study using the validation group. The AUC of the nomogram in the validation group was 0.819, demonstrating good predictive ability (**Figure 3B**). On the calibration graph, the model had good calibration in the validation group (Both p value > 0.05 in training and validation cohorts, respectively) (**Figure 4B**). Representative examples of TNBC-MC and TNBC-NMC are given in **Figure 5**. The decision curve analysis indicated that the nomogram model has good practicability (**Figure 6**).

### Follow-up information

**Figure 7:**
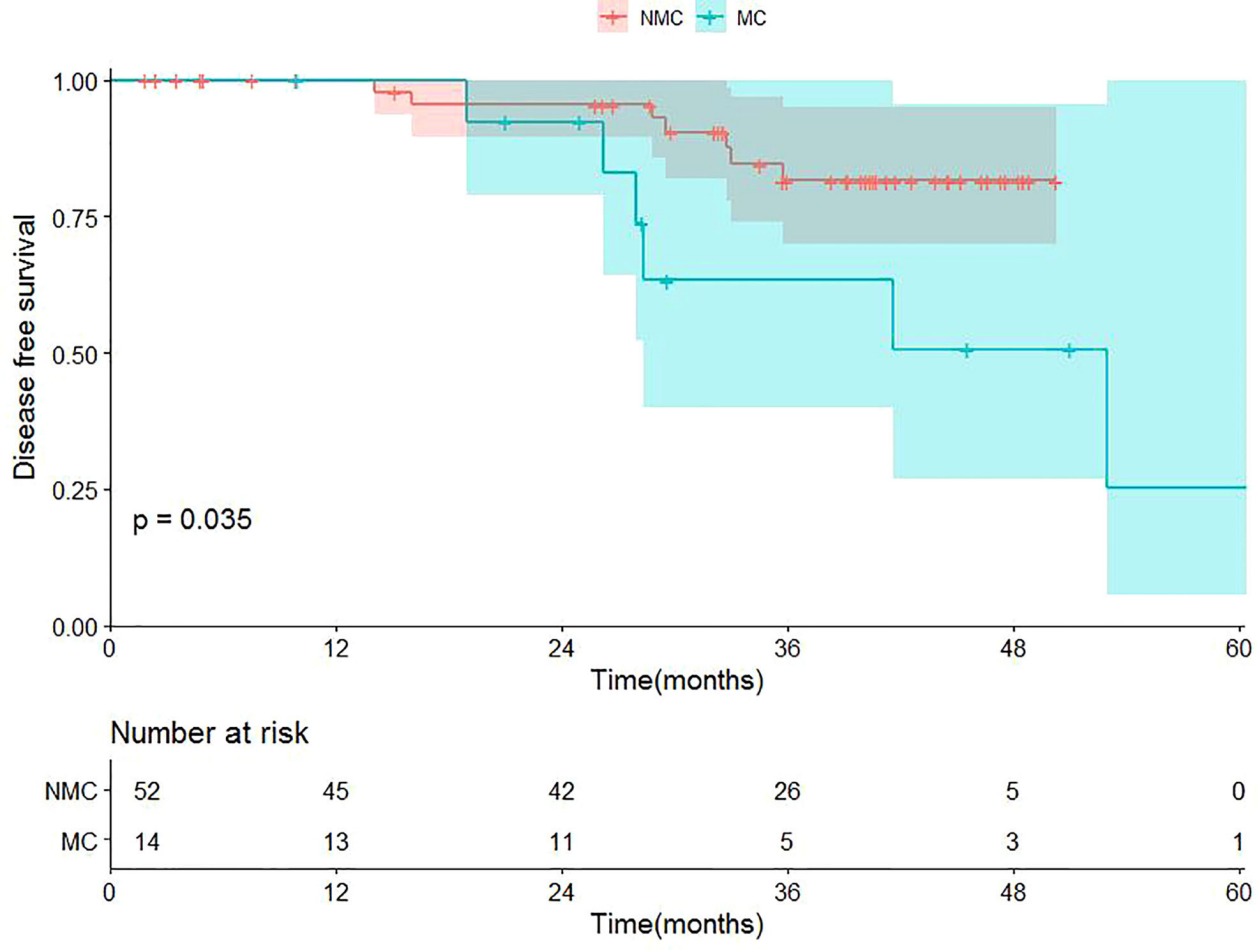
Kaplan-Meier survival curves of the cohort. TNBC-MC (abbreviation: MC), triple negative breast cancer-metaplastic carcinoma; TNBC-NMC (abbreviation: NMC), triple negative breast cancer - non metaplastic carcinoma.

Up to the cutoff date for this analysis, 6 of 14 TNBC-MC patients experienced recurrence, while 7 of 52 TNBC-NMC patients experienced recurrence. The median survival time (MST) of TNBC-MC was 849 days, while the MST of TNBC-NMC was 1077 days. Of 14 TNBC-MC patients, 6 experienced locoregional recurrence. Of these, two relapses occurred in the chest wall, two in the ipsilateral breast, one in the lung and one in the bone. Of 52 TNBC-NMC patients, 7 experienced locoregional recurrence. Of these, two relapses occurred in the chest wall, two in the ipsilateral breast, two in the lung, and one in the bones. The DFS of the two subtypes was significantly different (p=0.035) (**Figure 7**).

## Discussion

TNBC is a heterogeneous disease with a variety of histological types and has been considered a molecular subtype of breast cancer with aggressive behavior and poor prognosis (23). Several recent studies have indicated that TNBC-MC appears to have very different clinicopathologic parameters compared with TNBC-NMC (24, 25). Our study validated the prognostic difference between TNBC-MC patients and TNBC-NMC patients. We retrospectively analyzed the clinical and MRI features of TNBC with different histological types and developed a predictive nomogram for noninvasively predicting TNBC-MC and TNBC-NMC. In our model, MRI variables, including lesion type and internal enhancement pattern, were independently associated with histological types of TNBC. Our nomogram showed good discrimination and calibration abilities in the validation cohort.

Most of the previously published studies that used MRI features to explore the intratumoral heterogeneity of breast cancer mostly focused on the molecular subtypes of breast cancer (26–30). However, the heterogeneity of TNBC can be derived from histology, and it is easy to apply in clinical work (31, 32). This study developed a nomogram based on multiparameter MRI variables, which can not only reflect the histological heterogeneity of TNBC but also are relatively simple and easy to apply.

In this study, some MRI features, including lesion types and internal enhancement patterns, were found to differ between TNBC-MC and TNBC-NMC. MC lesions present as mass enhancement on enhanced MRI, which was consistent with our finding (12, 33, 34). In this study, most TNBC-MC patients (96.6%) presented mass enhancement. Most TNBC-NMCs are invasive breast carcinomas of no specific type (IBC-NST). Several studies have shown that IBC-NST of TNBC most often presents as mass enhancement (82%-95%) (35–38). In this study, only 44.3% of TNBC-NMCs presented mass enhancement, which was presumed to be caused by our more detailed classification of lesion types into mass enhancement and nonmass enhancement & both. However, this detailed classification is more helpful for distinguishing TNBC-MC and TNBC-NMC, and there is a significant difference between the two, so the lesion type was included in this nomogram model.

Many previous studies have reported that the most common internal enhancement patterns of MC are heterogeneous enhancement and rim enhancement, which corresponds to central necrosis and enhanced peripheral solid portions (12, 39, 40). Jia et al (39) reported that more than half (58.3%) of MCs show rim enhancement, similar to the results of this study (52.4%). In this study, although rim enhancement could be seen in both TNBC-MC and TNBC-NMC, it accounts for more than half of the TNBC-MCs (51.7%) and a minority of the TNBC-NMCs (25.0%), which showed mostly heterogeneous enhancement (75.0%). As the internal enhancement patterns of TNBC-MC and TNBC-NMC partially overlapped, in this study, our nomogram model combined the internal enhancement pattern and lesion type and had high predictive ability. The AUC in the validation set reached 0.819. All the variables used in the nomogram were based on noninvasive MR factors, indicating that our nomogram model is easy to operate and can be easily put into practice.

The reason for the significance of pathological grade in univariate analysis might be that high pathological grade was more common in TNBC-MC patients compared with TNBC-NMC patients in this study, which was consistent with the previous literature (24, 41). MC patients tended to have a larger tumor size, lower lymph node invasion rate, higher tumor grade (42). Pathological grade was not significant in the multivariable analysis, possibly because of collinearity and correlation of some of these significant factors among themselves.

As it has been reported that MC is more aggressive than TNBC-NMC in clinical practice, this study further verified the prognosis of TNBC-MC and TNBC-NMC (24, 32, 41, 43, 44). Our study reviewed 14 TNBC-MC patients and 52 TNBC-NMC patients and compared three-year survival data between the two groups. Similar to previous findings (24, 43), our study showed that TNBC-MC patients tended to present a worse DFS than TNBC-NMC patients (p=0.035). This finding suggests that although TNBC-MC is also a subtype of TNBC, it differs from TNBC-NMC and has worse biological behavior and worse long-term clinical outcomes. This further confirms the heterogeneity and prognostic difference of TNBC. In this study, potential heterogeneity information was captured by MRI features, and the nomogram prediction model we constructed has good performance and is convenient for clinical application.

Nevertheless, there are some limitations in our study. First, as this study was a multicenter retrospective study, the MR instruments were not unified, and neither was the scanning sequence for the breast; however, these factors do not affect our diagnosis. Moreover, we obtained an abundant number of patients for a retrospective review and a more comprehensive analysis of MRI performance. Second, we did not evaluate the use of radiomics or texture analysis. Further study is needed to determine whether the ability of the nomogram could be improved if the nomogram is combined with radiomics or texture analysis.

In conclusion, our nomogram model incorporating lesion type and internal enhancement pattern was able to distinguish between TNBC-MC and TNBC-NMC using a noninvasive examination. Our nomogram model could be a valuable tool for estimating the histological heterogeneity of TNBC and further predicting the risk of malignancy.

## Data availability statement

The data analyzed in this study is subject to the following licenses/restrictions: Patient privacy. Requests to access these datasets should be directed to YG, eyguoyuan@scut.edu.cn.

## Ethics statement

The study involving human participants was reviewed and approved by Guangzhou First People’s Hospital of ethics committee. The patients provided written informed consent to participate in this study.

## Author contributions

Q-CK, W-JT, and S-YC contributed to conception and design of the study. Q-CK and W-JT wrote the manuscript. S-YC and W-KH analyzed the date. YH and Y-SL validated the data. Q-QZ and Z-XC searched the data. DH and JY proposed the methodology. YG edited the manuscript. All authors contributed to the article and approved the submitted version.

## Funding

National Natural Science Foundation of China (No. 81901711) The Special Fund for the Construction of High-level Key Clinical Specialty (Medical Imaging) in Guangzhou, Guangzhou Key Laboratory of Molecular Imaging and Clinical Translational Medicine.

## Conflict of interest

The authors declare that the research was conducted in the absence of any commercial or financial relationships that could be construed as a potential conflict of interest.

## Publisher’s note

All claims expressed in this article are solely those of the authors and do not necessarily represent those of their affiliated organizations, or those of the publisher, the editors and the reviewers. Any product that may be evaluated in this article, or claim that may be made by its manufacturer, is not guaranteed or endorsed by the publisher.
